# Diagnosis and initial management in psoriatic arthritis: a qualitative study with patients

**DOI:** 10.1093/rap/rkz022

**Published:** 2019-08-26

**Authors:** Emma Dures, Clive Bowen, Mel Brooke, Jane Lord, William Tillett, Neil McHugh, Sarah Hewlett

**Affiliations:** 1 Department of Nursing & Midwifery, University of the West of England; 2 Academic Rheumatology, Bristol Royal Infirmary, Bristol; 3 The Psoriatic Arthritis Support Group (PsAZZ), Bristol; 4 Royal National Hospital for Rheumatic Diseases; 5 Department of Pharmacy & Pharmacology, University of Bath, Bath, UK

**Keywords:** qualitative, diagnosis, psoriatic arthritis, rheumatology, specialist care, psychological distress, self-management, treatment decisions

## Abstract

**Objectives:**

PsA is an inflammatory condition that can cause pain, fatigue, swelling and joint stiffness. The consequences include impaired physical function, a high psychosocial burden, reduced quality of life and work disability. The presenting symptoms can be non-specific and varied, leading to delays in diagnosis or referral to specialist teams. The aim of this study was to explore patients' experiences of being diagnosed and the initial management of PsA.

**Methods:**

The study used a qualitative design, with data collected in one-to-one, face-to-face semi-structured interviews.

**Results:**

Fifteen newly diagnosed patients (<24 months) from three hospital sites in the southwest of England participated. Interviews were transcribed, anonymized and analysed using inductive thematic analysis. The following two main themes with sub-themes represent the data: symptom onset to specialist care: ‘it was the blind leading the blind’ (making sense of symptoms; mis-diagnosis and missed opportunities; and fast and easy access to expertise); and diagnosis as a turning point: ‘having somebody say you've got something wrong with you, I was euphoric’ (validation and reassurance; weighing up treatment options; taking on self-management; and acknowledging loss and change).

**Conclusion:**

Participants were already dealing with functional limitations and were highly distressed and anxious by the time they received their diagnosis. Physical and mental outcomes could be improved by the implementation of existing psoriasis management guidelines and strategies for earlier referral from primary care to rheumatology and by the development of guidelines on educational, self-management and psychological support provision soon after diagnosis.

**Key Messages**

- Implementation of existing psoriasis management guidelines could reduce delays from symptom onset to rheumatology referral.
- At diagnosis, patients already experienced functional limitations, distress and anxiety.
- Educational and psychological support soon after diagnosis could help patients manage the impact and engage with self-management.

## Introduction

Psoriasis is a long-term, inflammatory skin condition characterized by erythematous papules (skin lesions) and plaques with overlying scale. A UK population-based cohort study concluded that around 2.8% of the general population have psoriasis [1]. PsA, an inflammatory arthritis with an estimated prevalence in the UK of 0.3–1.0% of the total population, is closely associated with psoriasis. PsA affects men and women equally, and its incidence peaks between the ages of 30 and 55 years [2]. It is a long-term condition, with flares of disease activity and periods of remission. Symptoms can include a red, scaly rash (psoriasis), swelling in the fingers and toes (dactylitis), stiff and painful joints, thickening and pitting of the nails, and fatigue. The impact of symptoms includes limited physical function, reduced quality of life and a high psychosocial burden [3]. On a societal level, PsA is associated with significant health-care costs, work disability and unemployment [4].

A systematic review and meta-analysis of observational and clinical studies estimated the prevalence of PsA in patients with psoriasis to be one in four [5]. However, diagnosis is complex. Although psoriasis precedes the onset of PsA in 84% of patients, a small number will develop the rheumatic condition before experiencing any skin symptoms, and some may never develop skin symptoms [6]. There is no definitive test for PsA. Diagnosis is usually made by rheumatologists based on clinical expertise and a combination of a process of elimination, medical history, physical examination, blood tests, MRI and X-rays of the affected joints.

The difficulty of diagnosis is important because the burden of undiagnosed PsA is high, and there is emerging evidence that delay in diagnosis results in worse outcomes [7]. Even a 6 month delay from symptom onset to the first visit to a rheumatologist contributes to the development of peripheral joint erosions and worse long-term physical function [8]. A systematic review and meta-analysis estimated the prevalence of undiagnosed PsA to be 15.5% [9]. Treatments for PsA have two main aims: to relieve symptoms; and to slow the underlying progression of the condition. Many pharmacological treatments are available for the management of PsA, including MTX and other synthetic and biological DMARDs [10]. Recent evidence suggests that patients who undertake early ‘aggressive’ treatment, with more frequent assessment and escalation in medication, can reach ‘low disease activity’ or even remission (the absence of signs or symptoms of disease activity) [11]. However, it is important to recognize that the condition can be clinically mild, requiring minimal or no pharmacological treatment.

The PROMPT programme (NIHR grant: RP-PG-1212-20007) is investigating the clinical and cost benefits of the early detection of PsA. The main study is a two-arm parallel-group cluster randomized controlled trial of screening, using enhanced surveillance for PsA in primary care *vs* standard care. The aim is to determine whether intervention leads to earlier detection of undiagnosed PsA, which in turn improves long-term outcomes for patients. Patients’ experiences and views of the current health-care context are likely to inform any implementation of a national screening programme. Currently, the range of factors contributing to delays in diagnosis are not well understood. They may include patient-related factors (e.g. reluctance to seek medical help with symptoms) and clinician-related factors (e.g. the heterogeneous nature of PsA and lack of autoimmune diagnostic markers, making diagnosis challenging) [12]. Evidence is needed of the patient journey from initial symptoms and interactions with health-care professionals, through to diagnosis and early management of PsA. The aim of this study was to gain insight into the experience of obtaining a diagnosis of PsA and how this impacted the engagement of patients with treatment and care.

## Methods

### Study design

A qualitative design was used because the study aimed to explore patients’ experiences and views. Data were collected using one-to-one, face-to-face, semi-structured interviews. Semi-structured interviews can generate insights into the thoughts, feelings and social worlds of participants [13]. In the semi-structured format, participants are asked the same core questions, but there is flexibility to probe more deeply and develop new lines of enquiry based on responses. The core questions in this study were based on an interview schedule designed by the research team, comprising patient research partners, rheumatology and nursing clinicians and qualitative methodologists (see Table 1).

**Table 1:** Core interview questions

A: initial symptoms	First symptoms noticed
	What these symptoms indicated
	Response to these symptoms
	Time before seeking medical advice
B: interaction with health-care professionals	Reasons for contacting a health-care professional/GP
	Health-care professional/GP response to symptoms
	Referral to rheumatology
	Initial interactions with rheumatology
	Thoughts and feelings about diagnosis
	Views on health care/care pathways
C: post-diagnosis	Treatment-related decisions
	Impact of PsA and treatment

GP: general practitioners.

The study was approved by the Proportionate Review Sub-committee of the North East – Newcastle & North Tyneside 1 Ethics Committee (reference: 16/NE/0393) and the Health and Applied Sciences Faculty Research Ethics Committee of the University of the West of England (reference: HAS.17.03.129).

### Sampling and recruitment

Participants were sampled from three diverse secondary care sites in the Southwest of England: a large, non-specialist hospital; a small, non-specialist hospital; and a specialist PsA centre. Recruitment was via two processes: eligible patients were either given study information by a research nurse, rheumatology consultant or researcher when they attended a hospital outpatient clinic; or they received study information through the post, based on the local clinical database. Patients were eligible to take part if they were >18 years old, had received a diagnosis of PsA confirmed by a rheumatology consultant within the previous 24 months, had enough English language to participate in an interview and had capacity to give informed consent.

### Data collection

Interviews were conducted in non-clinical rooms at the secondary care site where the participant was under the care of the rheumatology team. Before the start of the interviews, participants signed a consent form and provided demographic data (age, sex, disease duration) and data on current medications and disability (HAQ) [14].

Interviews were conducted by the lead author, who had no prior relationship with the participants. Interviews were audio-recorded, transcribed by a professional transcription service, checked for accuracy against the original audio files, and anonymized by changing the names of people and places.

An inductive thematic analysis was conducted [15]. This approach was selected because it is appropriate for research that is not based on pre-existing theory about the phenomena being studied. It is suitable for highlighting both similarities and differences across participants [16]. This is important because we aimed to capture variation in experiences of patients newly diagnosed with PsA. The lead author read all 15 transcripts and coded chunks of text that related to the research topic. Related clusters of coded text formed sub-themes, which were grouped together to form a smaller number of higher-order themes that described broad, often abstract, elements in the dataset. A sub-set of six anonymized transcripts were analysed independently by four members of the study team (three patient research partners and a clinical academic) to ensure that the findings were informed by multiple viewpoints [17]. The final analysis was based on the integrated interpretations of these five team members.

## Results

Fifteen patients participated in the study (10 women and 5 men). They ranged in age from 21 to 73 years and had received a diagnosis of PsA between 1 and 24 months before taking part in the study (see Table 2).

**Table 2:** Demographic and clinical data

Gender	**Age** ^a^	TDX	Medications	**Flare** ^b^	**HAQ** ^c^
M	51	6	MTX	Y	2.625
M	72	6	MTX; folic acid	NS	0.875
F	66	12	SSZ; paracetamol plus codeine	Y	1.75
F	32	24	Zapain, SSZ; LEF, MTX	Y	0.125
F	71	1	None	Y	1.5
F	61	15	paracetamol; tramadol; omeprazole	Y	2
F	48	3	MTX, zapain, ibuprofen	N	1.5
F	53	4	MTX; folic acid	Y	0.875
M	64	1	CSs; (due to start MTX + folic acid)	N	0.75
F	57	15	SSZ; MTX; prednisolone; naproxen; omeprazole	N	1.5
F	21	16	Adcal – D3	N	0
M	55	NS	None	N	0
F	36	18	SSZ; omeprazole	N	0.875
F	48	12	MTX; folic acid; fentbid forte gel	Y	0
M	73	2	Voltarol	N	0.125

a In years.

b Self-report of current active disease (N: no; NS: not sure; Y: yes).

c Heath Assessment Questionnaire: disability index of daily physical activities, with scores ranging from zero (without any difficulty) to three (cannot be done at all).

TDX, time since diagnosis, in months.

Two main themes represent the data, and each theme has three parts: the label; the theme summary; and the sub-themes. Theme labels and sub-themes are listed in Table 3. Data excerpts evidencing sub-themes are in Tables 4 and 5.

**Table 3:** Labels of main themes and sub-themes

Main theme	Sub-themes
1. From symptom onset to specialist care: ‘it was the blind leading the blind’	Making sense of symptoms
	Mis-diagnosis and missed opportunities
	Fast and easy access to expertise
2. Diagnosis as a turning point: ‘having somebody say you’ve got something wrong with you, I was euphoric’	Validation and reassurance
	Weighing up treatment options
	Taking on self-management
	Acknowledging loss and change

**Table 4:** Data excerpts evidencing theme 1 sub-themes

Theme 1: symptom onset to specialist care: ‘it was the blind leading the blind’
(i) Making sense of symptoms
When my hands started swelling, yes that’s when I began to think maybe I should do something about it, because they were getting quite painful and I was having more trouble doing things (F, 71 years old)
I’ve put my body through such a lot, and now it’s kicking me and telling me I’m getting my own back (M, 56 years old)
I wasn’t sure if it was stress related because I was going through a divorce at the time (F, 36 years old)
I think it was the blind leading the blind for a little bit. The doctor going ‘I don’t know’ and me going ‘well I don’t know’ (F, 32 years old)
I started mapping out a life plan of everything I did, my diet, what time I was getting up, absolutely everything, and I couldn’t see a pattern, it didn’t seem to be linked to exercise or anything, it didn’t seem to flare up when I did more or less (M, 55 years old)
I did have three blood tests for RA with the doctor, so it wasn’t as though nothing was done (F, 61 years old)
Because I wasn’t getting anywhere fast, and because it had had such a massive impact on my lifestyle, I actually felt I didn’t want to live (F, 48 years old)
Every day I’d wake up with a different joint, and I felt very depressed about it, very miserable, I stopped going out, I stopped socializing, and I felt like I was becoming disabled (F, 36 years old)
I couldn’t hold a cup at one point, and to go from being perfectly alright, to being like that in a space of a couple of months, I just wanted to kill myself, I really did (F, 48 years old)
(ii) Mis-diagnosis and missed opportunities
I went to the doctor [GP] and it was only in my, just above my thumb and they said it was RSI [repetitive strain injury] (F, 53 years old)
After examination and everything, he thought that I had OA (F, 66 years old)
… Oh you know, attention-seeking menopausal woman … I feel a bit cross really that they didn’t take it a bit more seriously, because I think how much damage has been done while I haven’t been getting any treatment (F, 57 years old).
It was opportunities missed, because the more I read about the inflammation, the systemic inflammation, the more scared I get, and I just want it under control (F, 48 years old)
A little bit angry, to be honest, that it wasn’t picked up for years, and possibly my fingers, which look deformed now, could have been stopped (F, 53 years old)
Every time I’ve been to have my creams and stuff you need for the psoriasis, none of them have ever assessed me for PsA, which they’re supposed to aren’t they, under the NICE guidelines, you’re supposed to be assessed every year (F, 48 years old)
I did read something on the NHS website that if you have psoriasis the doctor’s surgery should check you for arthritis once a year (F, 53 years old)
I remember going to the doctor and saying, because I have got psoriasis do you think it’s connected because I have got, my nails are a bit ridged (F, 57 years old)
This type of arthritis is normally seen in somebody that would suffer with psoriasis. Now, I don’t, externally. My mum did and my sister does, and my sister has gone on to have systemic lupus (F, 48 years old)
Well, I know my grandson has it [PsA], and we were all quite worried about it (M, 72 years old)
Members of my family do have PsA, so it was family history in the end (F, 21 years old)
(iii) Fast and easy access to expertise
I literally went from having a bit of a sore finger, saw a nurse, saw a doctor, went to the hospital, and it happened really quickly, and I was really happy with it (F, 21 years old
I haven’t been messed about or anything, the original GP had said right I will do this for you, which he did … so, yeah, I think it’s been really good. I have been treated well (M, 64)

F: female; GP: general practitioner; M: male.

**Table 5:** Data excerpts evidencing theme 2 sub-themes

Theme 2: diagnosis as a turning point: ‘having somebody say you’ve got something wrong with you, I was euphoric’
(i) Validation and reassurance
Having somebody say, yeah, actually you’ve got something wrong with you, you are in trouble, and I was euphoric at getting, being given a diagnosis (F, 57 years old)
Knowing what it was was definitely a big help to me and not knowing what it was was a source of great stress (F, 61 years old)
When you see a specialist and someone deals with you, it is, it’s reassuring (M, 51 years old)
I feel they want to put a label on it. I’m not wholly in agreement they’ve got it right (M, 55 years old)
I do feel now I’ve got to a specialist department, I feel confident that they’re going to do their best for me, as long as I do my bit and do my best myself (F, 57 years old)
Having this diagnosis hasn’t stopped the condition, yes, it’s opened up doors for treatment that hopefully will control it (F, 48 years old)
It’s [symptoms] still happening and they’ve upped the dosage to try and beat it, but I’ve got confidence at least it will, it won’t be through the lack of trying anyway (M, 51 years old)
It’s the emotional support, it’s incredibly important. I think it would be quite easy just to go under with it, and just to end up off work for weeks with depression, because it is depressing, because it affects everything (F, 36 years old)
It’s like, how could it go from my toe to taking MTX? It’s, yeah, it’s been very, very hard to get my head around. That’s what I mean about the whole emotional and mental health side of things. There’s nobody there you can talk to about it, because your doctor kind of like, ‘oh well it’s just monthly blood tests’ (F, 48 years old)
(ii) Weighing up treatment options
He [rheumatologist] said ‘it's your decision whether you want to take strong medication or not’ and I said I'd rather not. It's not bothering me that much, and he seems to agree with my decision. He doesn't think I'm being silly (M, 73 years old)
It’s been a discussion always, so I would do research, they have their own knowledge and own experience and then we’d collaborate and make decisions together (F, 21 years old)
Everything was explained to me really well. I came in, like, not knowing a thing. I come out not an expert but understanding, you know, what the problem was and stuff (M, 64 years old)
The fact that they are going to check liver and kidneys and things like that in blood tests makes it quite scary, because you think obviously these side-effects could be quite, not dangerous, but serious (F, 71 years old)
I found it very difficult, so I thought, okay, I need to be in the right place to reduce my alcohol intake mentally and I then need to decide if taking MTX is going to make me ill or not and is it worth it as I’ve lived with this so long already but I don’t want it to get worse (F, 53 years old)
I wouldn’t get to the point where I was completely immobilized by it or let the disease damage the joints. I wouldn’t go that far. I would prefer to control it with diet and exercise if there’s a way of doing that (M, 55 years old)
I was on MTX. It wasn’t doing an awful lot of good, so they upped the dose from 15 to 20 and then my liver started playing up. My liver was not working, my hair started falling out, all that sort of thing, so they put me back down to 15, and then they started me on, I can’t remember, LEF (F, 48 years old)
(iii) Taking on self-management
You can do something about it yourself, you know, as regards taking the medication and also doing the exercises, which physio will tell you what to do (M, 72 years old)
There was a lot I found I could do, and I’ve lost a lot of weight in the last year. And that’s helped a lot as well. So, I think you just sort of have to know what you can do yourself (F, 32 years old)
What I have found helpful, throughout all this, I have tried to meditate, and I was given a relaxation tape, which I do find very helpful (F, 61 years old)
That particular appointment was a total waste of time and just left me feeling very depressed and guilty. It makes you feel guilty, as if you’re doing something really wrong all the time (F, 66 years old)
(iv) Acknowledging loss and change
I had to pack up gardening for a start because I usually go to what I call gardening club on a Thursday, and that’s usually quite physical work. I can’t grip, so I don’t go (M, 72 years old)
Still more time off work. Now it’s starting to impact me financially (F, 48 years old)
I do worry now that employers are not going to want to employ me (F, 21 years old)
I’ve got an elderly mother and I feel so guilty that I can’t help her with anything (F, 61 years old)
I am very worried about when it [baby] does arrive, what’s going to happen, I don’t want everything to fall on my partner. He’s been incredibly supportive, but the fear is he will end up becoming my carer (F, 36 years old)
I have given up on the hope that my life will ever return to what it was because I feel that I’ve got to be realistic.… But if this is as good as it gets then I need to make a life that’s worth living (F, 48 years old)
Once you’ve got it, you’ve basically just got to learn to live with it and control it and do your best really, just accept it. Some things you can’t change (F, 66 years old)
I’m still really up and down with it. And there are times when I feel very bleak and depressed about it. And other times where I’ve got more get up and go, and that’s usually when I’m in less pain and less tired (F, 36 years old)

F: female; M: male.

### Theme 1: symptom onset to specialist care: ‘it was the blind leading the blind’

The time taken to obtain a referral from primary to secondary care varied between participants. During this period, participants and their general practitioners (GPs) tried to make sense of intrusive and often perplexing symptoms. Participants who spent longer with symptoms but without a diagnosis often struggled more with the long-term emotional impact than those who were referred to rheumatology departments after only a few GP visits. This struggle was attributed to high levels of pain, fatigue, the increasing impact of symptoms on daily life, loss of confidence in health-care professionals and high levels of anxiety about the future (see Table 4).

#### Making sense of symptoms

Participants presented at GP consultations with symptoms such as stiffness, swelling, stinging, hot burning sensations, and pain in their hands, wrists, ankles, feet or knees. Often, it was an unacceptable level of pain combined with an inability to carry out routine daily tasks that prompted participants to see a GP.

Participants’ initial beliefs about the causes of the symptoms included: over-doing a physical activity (e.g. gardening or typing); psychological stress (e.g. owing to divorce, house renovations or work relocation); an inevitable aspect of ageing; consequences of childhood physical activity (e.g. dance); acute sports injuries; and body changes post-pregnancy or during the menopause. These beliefs often contributed to an initial delay in contacting a health-care professional, because participants thought the symptoms would resolve without intervention. For some, symptoms seemed to come and go at random, resolving on their own only to flare up again sometime later. Participants looked for patterns related to their daily activities, diet and exercise, but were still unable to work out what was happening and why. Often, GPs also found it difficult to make sense of symptoms and piece together what was happening. This difficulty was compounded in some cases where a blood test came back as ‘normal’.

Over time, the experience of worsening, debilitating symptoms and not knowing why took an immense psychological toll. Participants described a life increasingly dominated by anxiety, pain and restriction. For some, the impact was overwhelming to the point of passive suicide ideation (expression of a wish to die, but without specific plans to commit suicide) during the interviews.

#### Mis-diagnosis and missed opportunities

Many participants reported multiple visits to health-care professionals, usually GPs, over a period of years before they received their diagnosis of PsA. During this time, there were often mis-diagnoses, which delayed referral to rheumatology, including repetitive strain injury, OA and Morton’s neuroma. This protracted journey could erode the confidence of participants in health-care professionals and the health-care system. The negative views of participants about the time spent seeking answers in primary care were compounded when they perceived that GPs had been dismissive or disbelieving, and they had sustained unnecessary joint damage as a result.

Participants acknowledged that GPs could not be expected to have specialist knowledge about PsA. However, they often believed that there had been missed opportunities at various points in the health-care system. This was particularly the case for patients with psoriasis who were under the care of dermatology teams. Subsequent to their PsA diagnosis, participants became aware that health-care professionals had not adhered to National Institute for Health and Care Excellence (NICE) guidelines for assessment of patients with psoriasis for PsA. Almost every participant explained that a family member had either psoriasis, an inflammatory rheumatic condition or both. For many, symptoms started to make sense and a diagnosis was reached when health-care professionals asked about family members.

#### Fast and easy access to expertise

Circuitous journeys to diagnosis contrasted with those participants who described fast and efficient GP referrals to rheumatology. As a result, these participants felt comparatively safe and supported, and satisfied with their health care.

### Theme 2: diagnosis as a turning point: ‘having somebody say you’ve got something wrong with you, I was euphoric’

Getting a diagnosis was a psychological and practical turning point for participants. This related to being believed and reassured by health-care professionals with the relevant expertise and coming to understand that their symptoms were attributable to a manageable long-term condition. The diagnosis enabled participants to act, such as starting treatments, and to make psychological adjustments, such re-evaluating their expectations for the future. However, for some the initial relief was short lived. Their sense that physical and psychological damage had already been done made them fearful for their long-term health. Despite engaging with their health care and making helpful lifestyle changes, some participants continued to experience high levels of emotional distress (see Table 5).

#### Validation and reassurance

After the route to diagnosis had been drawn out and participants had felt dismissed or discredited, diagnosis was experienced initially as a relief and a form of validation. It made sense to participants, except for one patient who wondered whether it reflected health-care professionals’ wish to assign a label, rather than certainty about PsA. Diagnosis also brought the possibility of managing the condition. Specialist medical expertise was a valued source of practical support, making participants feel confident and hopeful about symptom improvement.

However, the relief at diagnosis could be short lived, because the condition and its treatment continued to impact participants’ daily lives. A lack of emotional support influenced the ways in which participants coped with their PsA, with several reporting low mood, depression and anxiety. They discussed how they would like to access resources to address these feelings. This was an aspect of care that was identified as lacking.

#### Weighing up treatment options

Diagnosis led to decisions about treatments and behavioural changes to manage the impact of PsA. Communication with the rheumatology team was a key factor in how participants worked through their mixed feelings about undertaking treatment. Participants often reported a process of shared decision-making with the clinical team, underpinned by good provision of information. Discussions about pharmacological treatments could alarm participants; for example, the association of MTX with cancer treatments and the need for blood monitoring. However, the possibility of long-term disability and joint damage was also a worry and influenced participants’ treatment decisions. At the time of the present study, some in this group of newly diagnosed participants were still struggling to find a treatment regimen that could help with their symptoms and control their PsA.

#### Taking on self-management

Alongside the medical management, participants discussed the importance of doing what they could to help themselves keep well. This included a range of behaviours to minimize or reduce the impact of PsA, such as exercising joints, pacing activity, relaxation techniques, weight loss, reducing alcohol intake with MTX, and keeping physically active. However, supporting self-management and lifestyle changes, such as smoking cessation, required health-care professionals to work with the patient, and to understand their priorities and resources at that point and to avoid blame or criticism that might cause upset or resistance.

#### Acknowledging loss and change

Participants reported multiple losses, including valued activities and their sense of pre-symptom onset identity. Some participants were coming to terms with financial losses and worries about prospects for paid work. There was upset and worry about not being able to fulfil important roles, such as caring for others, and the impact of this on other people. Accepting a future with PsA was described as challenging and an on-going process that takes time. For some participants, the emotional and psychological impact of PsA remained a continual challenge.

## Discussion

These findings have identified challenges and opportunities for intervention from symptom onset to treatment and care by rheumatology teams. Although participants acknowledged the difficulties facing GPs owing to the disparate and perplexing presentation of their symptoms, they also reported examples of being dismissed or disbelieved, and how the impact that symptoms were having on their psychological well-being was overlooked. Often, they were not asked about a family history of skin and rheumatic conditions, which was a key indication that their symptoms could be attributable to PsA. In addition, some participants reported a failure to adhere to NICE guidelines on the management of psoriasis (Clinical guideline CG153, updated in 2017 [18]. The guidance on assessment and referral states that people with any type of psoriasis should be assessed for PsA (Section 1.2.1.1)). One potential route to implementation of an educational or public health intervention for earlier diagnosis could be through the community pharmacy, with staff offering screening and information [19].

Diagnosis was a turning point for participants, often bringing relief and a sense of validation. PsA can vary in clinical severity, and how this was communicated in discussions about treatment options was important. Discussions between patients and members of the rheumatology team about whether to start medication and how to support self-management highlight that the need for an early diagnosis goes beyond the medical model. It includes the psychological need for an explanation and reassurance, sometimes after years of living with symptoms but with no diagnosis or effective treatment.

By this point, several participants were highly distressed and anxious. This has implications for patients’ physical and mental outcomes. There is a well-established body of evidence from inflammatory rheumatic conditions, such as RA, that the impact of negative affective states is far reaching; this includes poor treatment outcomes, poor adherence to medications, high levels of pain and fatigue, and poor social and sexual function [20–23]. There is less research available on psychological status specifically in patients with PsA, but emerging evidence suggests that psychological distress remains an issue long after the period around diagnosis. A qualitative interview study with 24 patients between 4 months and 29 years post-PsA diagnosis reported that suicidal ideation was commonly expressed [24]. Participants’ psychological struggles were attributed to fears about long-term physical deterioration and the strain of hiding their distress from other people, who they perceived as dismissive and belittling. Illness beliefs have been identified as an important factor in shaping patients’ responses to their PsA and the subsequent impact of living with the condition. In a sample of 179 patients with PsA, hierarchical multiple regression models indicated that negative beliefs about the consequences of PsA and behavioural disengagement as a coping method predicted levels of depression, after controlling for the severity of the condition [25]. The role of illness beliefs was further supported in a secondary analysis of data collected in eight focus groups with 41 participants with PsA [26]. In addition, the authors highlighted the influence of uncertainty about the condition and its management on how patients engaged with treatment-related decisions.

Taken together with findings from the present study, there is a strong argument for addressing the illness beliefs and coping strategies of newly diagnosed patients, an aspect of care that participants identified as missing. Support should include exploration of expectations about long-term outcomes and treatment efficacy. There is evidence that attention should be given to ‘illness uncertainty’, which can be defined as the inability to determine the meaning of illness-related events, shaped by ambiguity about the condition, complexity in relationship to treatment and the health-care system, lack of information about diagnosis or severity, and the unpredictability of the prognosis [27]. This uncertainly can be experienced as a cognitive stressor, a sense of loss of control, and a perceptual state of doubt, and has been associated with difficulties in adjusting and unhelpful coping styles [28].

The present study supports recent research looking at outcomes important to patients when designing and evaluating care pathways. Treatment of PsA is challenging because several health domains can be involved. In current clinical practice, flare is a measure of swollen or tender joint counts. However, a qualitative study found that social withdrawal, psychological symptoms, fatigue and loss of function were also significant features for patients [29]. A focus group study investigating treatment outcomes found that in addition to symptom alleviation, patients valued a reduction of disease impact, improved prognosis, and the minimization of treatment harm and burden [30]. Incorporating the assessment of these outcomes in clinical practice might facilitate discussions between patients and rheumatology teams and enhance patient well-being.

### Strengths and limitations

A strength of this study is that participants were reflecting on their experiences of diagnosis within the first 2 years, and in the current health-care system. Participants of a wide age range of took part, from 21 to 73 years old, with a range of PsA severity. Their data were analysed by team members with different backgrounds, including patient partners with first-hand experience of living with PsA, and academics and methodologists who work in rheumatology settings. A study limitation is that although the sample were recruited from three diverse sites, the experiences of participants were of health-care provision in the Southwest of England. In addition, future research should investigate the impact of gender and ethnicity on experiences of diagnosis and early management in PsA.

## Conclusions

The process from symptom onset to diagnosis was often difficult and drawn out, with participants and GPs unable to make sense of presenting symptoms. It seems likely that referral to rheumatology was delayed because questions were not asked about skin and rheumatic conditions among family members, and psoriasis management guidelines were not adhered to. Receiving a diagnosis could bring relief and was a practical and psychological turning point for some participants. The knowledge that they had PsA enabled them to consider treatment-related decisions and engage with a range of self-management behaviours. However, by this point many participants were also dealing with functional limitations and were highly distressed and anxious. A key feature of their distress related to uncertainty and fearfulness about the long-term impact of the condition. Care pathways could be enhanced by: implementation of existing psoriasis management guidelines and strategies to facilitate earlier referral from primary care to rheumatology; the development and implementation of guidelines for educational and self-management support at the point of diagnosis; psychological support provision for patients experiencing distress and anxiety to develop helpful coping strategies; and the evaluation of outcomes important to patients in care pathways. Basing treatment and care on such a biopsychosocial model has the potential to improve physical and mental outcomes for patients.

*Funding*: This report is independent research funded by the National Institute for Health Research, Programme Grants for Applied Research [Early detection to improve outcome in patients with undiagnosed PsA (‘PROMPT’), RP-PG-1212-20007].

This report presents independent research commissioned by the National Institute for Health Research (NIHR). The views and opinions expressed by authors in this publication are those of the authors and do not necessarily reflect those of the NHS, the NIHR, NETSCC, the Programme Grants for Applied Research programme or the Department of Health. The views and opinions expressed by the interviewees in this publication are those of the interviewees and do not necessarily reflect those of the authors, those of the NHS, the NIHR, MRC, CCF, NETSCC, the Programme Grants for Applied Research programme or the Department of Health.

*Disclosure statement*: The authors declare no conflicts of interest.
